# Disequilibrium calorimetry for determination of ultrasonic power in sonochemistry

**DOI:** 10.1016/j.mex.2017.08.003

**Published:** 2017-08-31

**Authors:** Mario Plattes, Christian Köhler, Tom Gallé

**Affiliations:** Luxembourg Institute of Science and Technology (LIST), Environmental Research and Innovation (ERIN), Luxembourg

**Keywords:** Calorimetry, Sonochemistry, Ultrasound

## Abstract

The two most characteristic properties of an ultrasonic wave are the frequency and the power. It is therefore important to determine the power in a given reactor. This can be done by calorimetry, i.e. by measuring the temperature rise in the vessel during sonication starting at thermal equilibrium with the surroundings (classic calorimetry) [1–3]. However, the classic ultrasonic calorimetry has drawbacks. In particular it is difficult to evaluate the temperature rise at thermal equilibrium, because the relevant initial time and temperature intervals are small and measurement errors in the temperature readings are large. Also the initial temperature response of the probe is complex [4]. The authors propose to start the calorimetric measurement at thermal disequilibrium, i.e. with a lower temperature in the reaction vessel. During sonication the temperature in the reaction vessel rises faster than in the surrounding and passes thermal equilibrium. The acoustic power transferred to the vessel at thermal equilibrium can then be calculated. The method consists of:

• Setting up the reaction vessel at lower temperature than the surroundings (ultrasonic bath or air).
• Measuring temperature rise in the reaction vessel and the surroundings during sonication.
• Determine the temperature rise at intercept by interpolation and calculate the ultrasonic power in the reaction vessel.

## Graphical abstract

**Figure:**
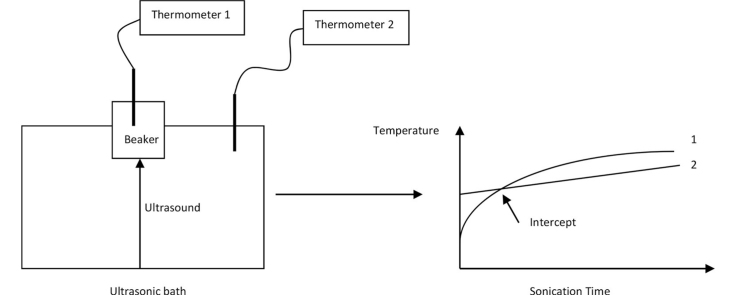

## Methods detail

**Fig. 1:**
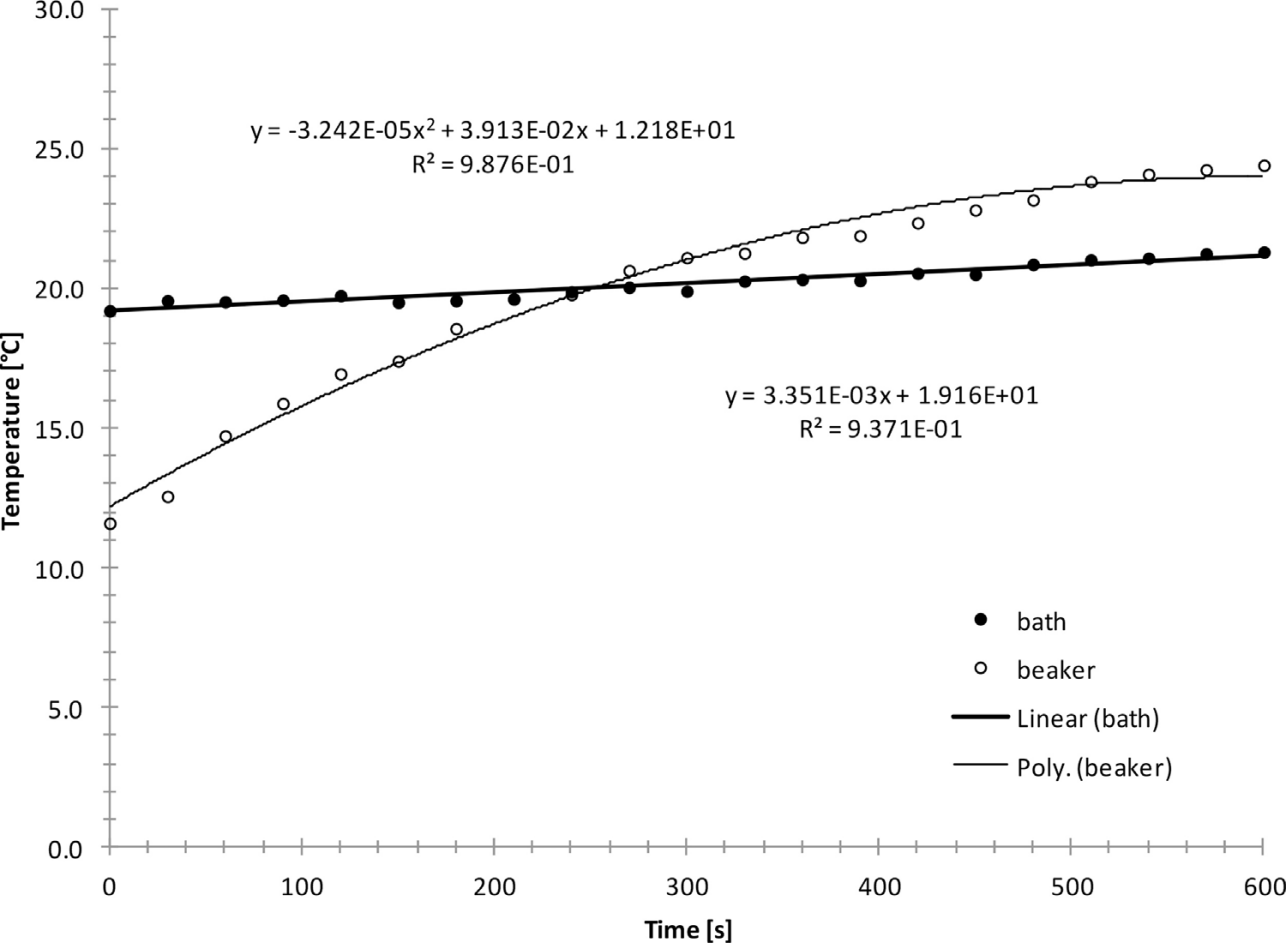
Typical temperature rises in the beaker and the ultrasonic bath during disequilibrium calorimetry.

A glass beaker (100 ml) was filled with 40 ml deionized water with a temperature lower than the temperature of the ultrasonic bath (VWR Ultrasonic Cleaner USC-TH, 45 kHz, 180 W). The beaker was then placed in the ultrasonic bath and the bath was switched on. The temperature rise in the beaker and the bath was recorded using two thermocouple type thermometers that had a precision of 0.1 °C (VWR Traceable Monitoring Thermometer). The experiment was repeated 5 times. A typical result is shown as example in Fig. 1.

It can be seen that the temperature rise in the beaker is curved and that it fits a polynomial of 2nd order. The temperature rise in the ultrasonic bath was linear and a straight line could be fitted to the data. The temperature rise in the beaker was faster than in the bath. The temperature in the beaker and the temperature in the ultrasonic bath intercepted at 20 °C after 250 s sonication. Note that it will take longer to reach thermal equilibrium if the initial temperature in the beaker is smaller, i.e. below 12 °C.

The slope of the temperature rise in the beaker was determined at intercept by interpolation and the power was calculated according to equation 1, where P is the power, c_P_ is the heat capacity of water (4.1813 J/g °C), m is the mass of water (40 g), T_1_ is the temperature in the beaker, and t is the time.

(1) $$P=c_{P}\times m\times {\left(\frac{dT_{1}}{\mathrm{dt}}\right)}_{\mathrm{Int}.}$$

The mean overall power in the beaker found after five experiments was 5.71 W (Table 1). The power density (W/cm^3^) and the intensity (W/cm^2^) are also given in the table below. The results show good reproducibility with a relative standard deviation (RSD) of 16%.

**Table 1:** Ultrasonic power determined by disequilibrium calorimetry.

Power			
	[W]	[W/cm^3^]	[W/cm^2^]
Mean	5.71	0.14	0.41
STDEV	0.89	0.02	0.06
RSD	0.16	0.16	0.16

The temperature rise in the beaker was faster than in the bath, because the power density (W/cm^3^) in the beaker was larger than in the bath. The reason for this is that the volume of the beaker was much smaller than the volume of the bath, although the intensity (W/cm^2^) at the center of the bath, where the transducer is attached, must be larger than at the bottom of the beaker

## Comparison with classic calorimetry

**Fig. 2:**
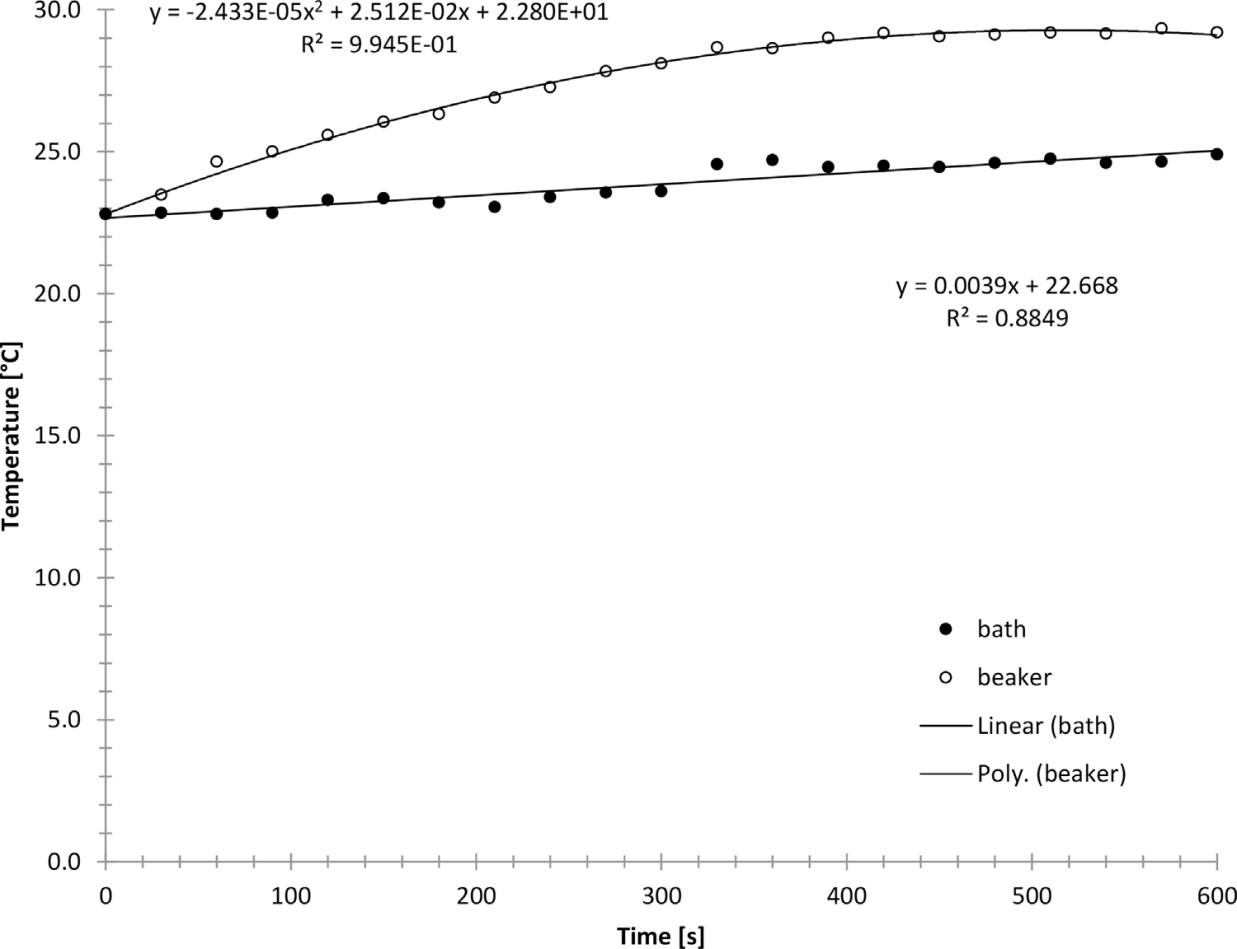
Typical temperature rises in the beaker and the ultrasonic bath during classic calorimetry.

A glass beaker (100 ml) was filled with 40 ml deionized water and placed in an ultrasonic bath (VWR Ultrasonic Cleaner USC-TH, 45 kHz, 180 W). The beaker was left in the ultrasonic bath until thermal equilibrium was reached. Then the bath was switched on and the temperature rise in the beaker (and the bath) was recorded using two thermocouple type thermometers that had a precision of 0.1 °C (VWR Traceable Monitoring Thermometer). A polynomial of 2nd order was fitted to the temperature rise in the beaker and the initial temperature rise at thermal equilibrium and time zero was evaluated. The ultrasonic power was then calculated using Eq. (1). The experiment was repeated 5 times. A typical temperature rise is shown in Fig. 2 and the resulting mean ultrasonic power and intensity after five experiments are given in Table 2.

**Table 2:** Ultrasonic power determined by classic calorimetry.

Power			
	[W]	[W/cm^3^]	[W/cm^2^]
Mean	4.20	0.10	0.30
STDEV	1.19	0.03	0.09
RSD	0.28	0.28	0.28

The ultrasonic power resulting from disequilibrium calorimetry (5.71 W) differs significantly from the ultrasonic power found by classic calorimetry (4.20 W). Further it was found that disequilibrium calorimetry is more reproducible (RSD = 16%) than classic calorimetry (RSD = 28%). We suggest that the reason for this that the point of thermal equilibrium is approached in the curve fitting and calculation from two sides in disequilibrium calorimetry, thereby minimizing measurement errors.

## Conclusions

• The ultrasonic power in a vessel can be determined by disequilibrium calorimetry.

• The resulting ultrasonic power has good reproducibility, i.e. a relative standard deviation of 16%, which is better than the relative standard deviation obtained by classic calorimetry, i.e. 28%.

• The mean ultrasonic power obtained by disequilibrium calorimetry (5.71 W) deviates significantly from the value obtained by classic calorimetry (4.2 W).

## Additional information and discussion

The part of the overall ultrasonic power that is transferred into heat is measured during calorimetry. Other proportions used for example for activation of reactants and bulk mixing are not taken into account by calorimetry. Still it is common practice to carry out calorimetric measurements in order to characterize sonochemical reactors [1], [2], [3]. Further the calorimetrically determined power will be different in other liquids, since the measurement is affected by vapor pressure and viscosity of the liquid [3]. Deionized water was chosen as medium because the measurements were carried out in the context of ultrasonic enzyme extraction from activated sludge, which is an aqueous medium. In addition, the shape of the reaction vessel will also affect the amount of ultrasonic energy transferred to the vessel, e.g. when a round bottom flask is used instead of a beaker.

The mean ultrasonic power obtained by disequilibrium calorimetry (5.71 W) differs significantly from the mean power obtained by classic calorimetry (4.20 W). We suggest the following explanation for this: During classic calorimetry cooling of the probe due to ultrasonic streaming takes place [4]. This cooling starts to manifest after a few milliseconds after switching on the ultrasound and results in a temperature decrease of the probe [4]. This cooling possibly results in a smaller temperature reading, in particular for the first measurement point after 40 s sonication, and thus a smaller evaluated power in classic calorimetry. This cooling effect does not take place during disequilibrium calorimetry at the point of interest, i.e. the point of thermal equilibrium. Therefore we believe that disequilibrium calorimetry provides the more representative result, since the effect of probe cooling due to ultrasonic streaming has been eliminated. Further reproducibility has been improved, since the point of thermal equilibrium is approached from two sides for ultrasonic power determination, thereby minimizing the measurement error introduced by the limited resolution of the thermocouple, i.e. 0.1 °C. The problem of reproducibility of results in sonochemistry has been stressed in the literature for other dosimetric methods, i.e. potassium iodide and methylene blue dosimetry [5]. The improved reproducibility is therefore an important advantage of the proposed method, i.e. disequilibrium calorimetry.

Disequilibrium calorimetry can also be applied to reaction vessels that are sonicated by an ultrasonic probe.
